# Spontaneous Fermentation‐Induced Changes in Selected Ghanaian Plant‐Based Flours: Physico‐Functional and Biochemical Insights for Complementary Food Use

**DOI:** 10.1002/fsn3.71740

**Published:** 2026-04-06

**Authors:** Richard Atinpoore Atuna, Fortune Akabanda, Jan Makurat, Ursula Bordewick‐Dell, Matthias Lamping, Guido Ritter, Francis Kweku Amagloh

**Affiliations:** ^1^ Department of Food Science and Technology University for Development Studies Tamale Ghana; ^2^ Department of Food‐Nutrition‐Facilities Muenster University of Applied Sciences Muenster Germany; ^3^ Centre of Competence for Humanitarian Relief Muenster University of Applied Sciences Muenster Germany; ^4^ Institute of Sustainable Nutrition Muenster University of Applied Sciences Muenster Germany

**Keywords:** cassava, cereals, color, phytic acid, plantain, proximate, soybean, sugar, sweetpotato

## Abstract

This study investigated the effects of spontaneous fermentation (SF) on the physicochemical and biochemical properties of seven Ghanaian staples: maize, millet, sorghum, sweetpotato, cassava, plantain, and soybeans. The cereals were soaked (12 h), ground, and fermented for 48 h. Cassava, plantain, and sweetpotato were peeled, grated, and fermented similarly. All samples were dried (3 days), milled into flour, and compared with unfermented controls. SF significantly enhanced water absorption in soybean flour and oil absorption in cassava, plantain, and sweetpotato flours. The foaming capacity generally declined, except for stable foams in millet and sweetpotato. The peak viscosity increased in most flours, except for cassava (−14%) and sorghum (−18%), following SF. There was an almost 3.6‐fold and 2.2‐fold increase in the trough viscosity of plantain and sweetpotato flours, respectively, after SF. Meanwhile, the final viscosity decreased in plantain (−72.6%) and sorghum (−20.6%) post‐SF. The crude protein content increased significantly in sorghum (11.3% vs. 10.5%) and soybean (41.2% vs. 39.6%) after SF treatment. After SF, the total polyphenolic content increased in maize (3.92 vs. 3.26 mgGAE/100 g), millet (5.05 vs 4.04), plantain (5.22 vs 4.91), and sweetpotato (29.7 vs 4.8), but declined in cassava (−18.5%), sorghum (−20.4%), and soybean (−13.8%). Phytic acid levels were generally reduced across all matrices, ranging from 23% to 69% after SF. These findings support the potential of SF to improve the nutrient density and functional properties of local staple foods. However, elevated polyphenol levels and final viscosity in some flours may affect nutrient levels and digestibility, necessitating careful formulation when developing complementary foods for infants and young children.

## Graphical abstract

Impact of spontaneous fermentation on functional, pasting, and biochemical properties of indigenous Ghanaian plant matrices.

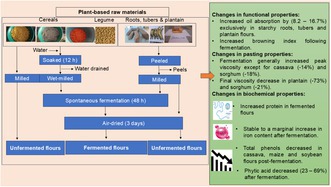

## 1 Introduction

Fermented foods have become an integral part of the cultural and traditional norms of indigenous communities in many countries, particularly in Africa (Chelule et al. 2010). Fermentation involves the enzymatic and microbial transformation of food substrates, usually resulting in desirable modifications in texture, flavor, and nutritional composition (Shiferaw Terefe and Augustin 2019). Cereal‐based fermented foods (CBFs) constitute a significant fraction of fermented products intended to alleviate food and nutrition insecurity in sub‐Saharan Africa (SSA) (Brandt 2014). The CBFs consumed in SSA are mainly cooked paste (*Tuo Zaafi, Kenkey, Banku*, and *Akassa*), porridge (*Mori Koko*), beverages (*Gowé* and *Mahewu*), cereal‐yoghurt (*Akpan*), and bread‐like products (*Aboolo, Kirsa*, and *Injera*) (Houngbédji et al. 2019; Mashau et al. 2021; Zannou et al. 2022). Although CBFs have been part of our cultural heritage, their recent surge in popularity can be attributed to their fast recognition as having a health‐promoting effect owing to enhanced nutrient bioavailability and the synthesis and accumulation of bioactive compounds (Adebo et al. 2022; Adebo and Gabriela Medina‐Meza 2020).

While the importance of fermentation cannot be overemphasized, the specific changes it induces in the techno‐functional and nutritional properties of cereals, legumes, and root tuber crops remain relatively understudied. Techno‐functionality refers to any property of food, aside from its nutritional value, that influences its utilization. Key techno‐functional properties essential in food processing include emulsification, solubility, water and oil binding, foam capacity and stability, gelation, and viscosity (Mygdalia et al. 2022; Shiferaw Terefe and Augustin 2019). The techno‐functional properties of ingredients directly or indirectly affect their processing applications, food quality, and overall acceptance and utilization in food product development (Mahajan and Dua 2002; Shiferaw Terefe and Augustin 2019).

The controlled fermentation of cereals and legumes has been reported to significantly influence their functional properties, including swelling capacity, dispersibility, emulsifying ability, and foaming capacity. (Mygdalia et al. 2022; Olukomaiya et al. 2020; Sadh et al. 2018). Despite the widespread use of spontaneous fermentation (SF) in traditional food preparation, relatively few studies have systematically examined its impact on the techno‐functional properties of cereals and legumes. For instance, Afoakwa et al. (2007) and Eshun et al. (2024) demonstrated that spontaneous fermentation enhances the functional properties of maize‐based cowpea‐fortified nixtamalized foods and cowpea flour. However, there is a dearth of information on how spontaneous fermentation influences the techno‐functional properties of commonly consumed staples in Ghana, particularly regarding their suitability as complementary food ingredients for infants. This study addresses this gap and provides insights that distinguish it from previously published studies on controlled fermentation.

Therefore, this study hypothesized that spontaneous fermentation (SF) would influence the techno‐functional and compositional profiles of selected grains, roots, tubers, and plantain flours. To test this hypothesis, this work aims to evaluate the impact of SF on the techno‐functional and biochemical properties of flours derived from Ghanaian maize, millet, sorghum, soybean, sweetpotato, cassava, and plantain. Understanding the impact of SF on diverse crops could inform the development of functional, value‐added products, potentially fostering new markets and economic prospects for farmers while providing additional nutritional benefits for complementary foods.

The selection of cereal grains is based on anecdotal and empirical evidence from Ghana, which consistently shows their use in the formulation of complementary foods at both the home and industrial levels (Suri et al. 2014). These cereals are usually mixed with protein‐rich foods, such as groundnuts and soybeans, in varying proportions to prepare a complementary food called weanimix (Lartey et al. 1999). Additionally, starchy staples such as sweet potatoes and cassava have been successfully incorporated into similar formulations (Amagloh et al. 2012; Wireko‐Manu et al. 2016). However, to the best of our knowledge, ripe plantains have not been investigated as complementary food ingredients. Therefore, their inclusion in this study represents a novel contribution to the field.

## 2 Materials and Methods

### 2.1 Sample Preparations

The samples used in this study included orange maize (Honanpa), pearl millet (Akadko'om), sorghum (Mankrazee), soybean (Favor), orange‐fleshed sweetpotato (JanLow), cassava (Abenwoha), and plantain (Bodipa). The grains and the legume were obtained from the Council for Scientific and Industrial Research, Savanna Agricultural Research Institute (CSIR‐SARI). Sweetpotato was obtained from a farmer in Damongo, Ghana, in the Savanna region. They were harvested at 3 months of age. Cassava and plantain were purchased in Techiman, Bono East Region, Ghana. The plantains were at stage 6 of ripening (all yellow), as categorized by Baiyeri (2005) for plantain and banana fingers. All samples were prepared using a standardized protocol adapted from Atuna et al. (2025b), ensuring consistency across diverse crop types while mimicking traditional processing steps relevant to complementary food preparation.

### 2.2 Experimental Design

The study employed a 2 × 7 factorial design, comparing two (2) processing conditions (unfermented and fermented) applied across seven crops: cassava, maize, millet, sorghum, soybean, plantain, and sweetpotato.

### 2.3 Functional Properties

#### 2.3.1 Water‐and Oil‐Absorption Capacity

The water and oil absorption capacities were determined following the procedures outlined in a previous study (Elkhalifa et al. 2005). Approximately 2 g of each flour sample was weighed in a pre‐weighed centrifuge tube. Approximately 20 mL of distilled water and 20 mL of vegetable oil were added to the WAC and OAC tubes, respectively. Samples were vortexed, incubated at ambient conditions for 30 min, and centrifuged at 4000 rpm for 25 min. Excess liquid, either water or oil, was decanted by inverting the tubes over absorbent paper and allowing them to drain. The weights of the water and bound oil samples were determined by difference. The hydrophilic–lipophilic index was expressed as the ratio of WAC to OAC, as reported by Medoua et al. (2005).

#### 2.3.2 Bulk Density

The method described by Abe‐Inge et al. (2020) was used to determine the bulk density. Ten grams of each flour type was weighed into a 50 mL graduated cylinder. For the loose bulk density, the sample was gently poured into the cylinder without compaction, and the initial volume was recorded. The measuring cylinder with the sample inside was gently tapped on the benchtop 10 times from a height of 5–8 cm. In both cases, the bulk density was determined and expressed in grams per milliliter (g/mL) based on the sample weight and corresponding volume.

#### 2.3.3 Foaming Capacity and Foam Stability

The foam capacity (FC) and foam stability (FS) were determined as described by Chandra and Samsher (2013). Approximately 1.0 g of each flour sample was added to 50 mL of distilled water in a graduated cylinder at 30°C. The suspension was mixed and shaken for 5 min to generate foam. The volume of foam at 30 s after whipping was expressed as foam capacity using the following formula:

$$\begin{array}{c} \text{Foaming capacity}\;\left(\%\right)\\ =\frac{\text{Volume of foam after whipipng}-\text{volume}\;\text{of foam before whipping}\;}{\text{volume of foam before whipping}}\;x\;100. \end{array}$$

The foam volume was measured 1 h after whipping to assess foam stability, which was expressed as a percentage of the initial volume.

#### 2.3.4 Dispersibility

Dispersibility was determined according to Kulkarni et al. (1991). Briefly, approximately 10 g of the flour samples was weighed into separate 100 mL measuring cylinders, and water was added to each until the 100 mL mark was reached. Then, the mixture was stirred vigorously for a short time; thereafter, it was allowed to stand for 3 h. The volume of settled particles was measured and subtracted from 100; the resulting difference was reported as the percentage of dispersibility.

#### 2.3.5 Swelling Index

The swelling index was determined using the method described by Ukpabi and Ndimele (1990). For each flour sample, 50 g was placed in a 500 mL graduated cylinder. Three hundred milliliters of cold water was added, and the mixture was allowed to stand for 4 h before the degree of swelling was observed. The swelling index was calculated as the ratio of the original volume to the final volume.

#### 2.3.6 Color Determination

The color of the samples was determined using a handheld chromameter (Model: Konica Minolta CR‐410), as described in a previous study (Atuna et al. 2023). Briefly, the chromameter was calibrated using a standard white tile. Flour samples from the various crops were placed in separate Petri dishes and covered. Chromameter measurements were performed by placing the lens at three separate locations on the sample. The color parameters recorded included L* (lightness, ranging from 0 = black to 100 = white), a* (negative values = green, positive values = red), and b* (negative values = blue, positive values = yellow). Chroma and hue angle values were also recorded. The Browning index (BI) was computed using Equation 1 below, as reported earlier by Kasim and Kasim (2015).

(1) $$\mathrm{BI}=\frac{100\;\left(x-0.31\right)}{0.17}.$$

where (in Equation 1) x; is calculated as.

(2) $$x=\frac{a*+1.75L*)}{\left(5.645L*+a*-0.012b*\right)\;}.$$

The total color difference (∆E) was calculated following the methodology of Kasim and Kasim (2015) using Equation 3.

(3) $$\left(\mathrm{\Delta E}\right)=\sqrt{{\left(\Delta a*\right)}^{2}+{\left(\Delta b*\right)}^{2}+{\left(\Delta L*\right)}^{2}}.$$

ΔE values were interpreted as follows: < 1.0 (imperceptible), 1.0–2.0 (perceptible upon close inspection), 2.0–3.5 (noticeable at a standard viewing distance), and > 3.5 (marked difference).

#### 2.3.7 Pasting Properties

The pasting properties of all flour samples were analyzed using a Rapid Visco Analyzer (RVA 4500; Perten, Australia) as described by Amagloh et al. (2013) and Atuna et al. (2021). Briefly, approximately 3.5 g (14% moisture basis) of each flour sample (in triplicate) was weighed and transferred to aluminium canisters. Approximately 25 mL of distilled water was added (corrected to account for 14% moisture in the sample). A plastic paddle was used to mix the slurry to break up any lumps before inserting the canister with the paddle into the RVA to measure the pasting profile for 13 min. The initial equilibration of the slurry was set to 2 min at 25°C, followed by heating to a maximum of 95°C for 5 min, and then holding at this temperature for a further 3 min. The paste was cooled to 25°C for 5 min and held at this temperature for 3 min. The maximum apparent viscosity during heating or during the heating/holding phase of the test was reported as the peak viscosity, and the final viscosity was recorded at the end of the test.

#### 2.3.8 Proximate Analysis

Proximate analysis was conducted in the Biochemistry and Chemistry laboratories at the Muenster University of Applied Sciences, Germany. The Official Methods of Analysis of the Association of Official Analytical Chemists (AOAC) International (AOAC 1995) were used to determine the moisture (AOAC 925.10), crude protein (AOAC 960.52), ash (AOAC 923.03), and crude fat (AOAC 922.06) contents. The total carbohydrate content was determined by difference. Thus, total carbohydrate = 100 – [moisture + crude protein + ash + crude fat].

#### 2.3.9 Total Energy

The Atwater general factor of 4–4‐9, as described by Merrill and Watt (1973) was used to compute the total energy of the flours from each crop before and after fermentation.

#### 2.3.10 Sugar Content

The maltose, D‐glucose, and sucrose concentrations in flours from various crops were quantified using an Enzytec Liquid Combi enzymatic assay kit (E8175), accepted as an AOAC official method of analysis (Lacorn and Hektor 2025).

### 2.4 Minerals

Mineral analysis was performed by the accredited laboratory GBA Gesellschaft für Bioanalytik mbH (Hamburg, Germany). The concentrations of minerals, including calcium, iron, potassium, magnesium, manganese, phosphorus, sulfur, and zinc, were determined by Inductively Coupled Plasma Optical Emission Spectrometry (ICP‐OES: Agilent 5110 ICP‐OES, Agilent Technologies, Santa Clara, CA, USA), as specified by the German official method 64 LFGBL 00.00–144 (ICP‐OES: 2019–07). Sample preparation and analysis were performed according to the prescribed protocol, and quantification was performed using certified reference standards. Zinc (Zn) analysis was conducted using Inductively Coupled Plasma Mass Spectrometry (ICP‐MS/MS: 8900 Triple Quadrupole ICP‐MS, Agilent Technologies, Santa Clara, CA, USA) in accordance with the modified standard DIN EN 15763 (ICP‐MS: 2010–04). Proper digestion processes and instrument settings were used to assess the precision and reproducibility.

### 2.5 Total Polyphenol

Each test sample underwent a sequential extraction protocol using acetone and water to isolate phenolic compounds. Approximately 0.25 g of the sample material was weighed and extracted in 25 mL of an 80:20 (v/v) acetone‐water mixture, maintained at 0°C in an ice bath with constant stirring. The solution was centrifuged at 4000 rpm at 4°C for 10 min, and the supernatant was transferred to a round‐bottom flask with a stabilizing reservoir of 1.25 mL of acetic acid. The remaining pellet was resuspended in 25 mL of a 60:40 (v/v) acetone: water mixture, stirred at ice temperature, and centrifuged again as described previously. The centrifugation and resuspension procedures were repeated. The three supernatants were combined and evaporated at 37°C under reduced pressure using a rotary evaporator.

The total polyphenol content (TPC) was quantified using the Folin–Ciocalteu assay on a microplate. Standards of gallic acid (20 mg/L, 40 mg/L, 60 mg/L, 80 mg/L, and 100 mg/L) were prepared in 2.5% acetic acid. A sample or standard (20 μL) was used with Folin–Ciocalteu reagent (10 μL), 40 μL of 10% sodium carbonate, and 130 μL of distilled water in each well of a flat‐bottom microplate. The plates were incubated at 70°C for 10 min and then cooled to room temperature. The absorbance of the sample was measured at 734 nm using a Spark multifunctional microplate reader (Tecan Austria GmbH, Grödig, Austria), and the TPC was determined from a gallic acid calibration curve.

### 2.6 Phytic Acid

The phytic acid levels in the samples were determined using the Megazyme Phytic Acid Assay Kit (K‐PHYT 05/19) (Megazyme, Bray, Ireland) according to the manufacturer's protocol, as reported previously (Grgić et al. 2023). The phytic acid concentrations in each sample were measured at 655 nm using a Spark multifunctional microplate reader (Tecan Austria GmbH, Grödig, Austria). Reference materials were included in each run to validate the assay performance. The percentage recovery of the phytic acid standards (oat flour) ranged from 85% to 98%.

### 2.7 Statistical Analysis

All statistical data processing was carried out in RStudio (version 4.5.1). A two‐way analysis of variance (ANOVA) was used to determine the interaction between crop type and fermentation on the measured parameters. The Fisher Least Significant Difference (LSD) test was used for post hoc comparisons when a significant interaction or main effect was observed. The data are presented as the mean (SD) from duplicate or triplicate determinations. A *p*‐value of 0.05 was selected as the threshold for statistical significance. All visualizations (e.g., bar plots and box plots) were created using ggplot2 and the associated tidyverse packages.

## 3 Results

### 3.1 Functional Properties

**FIGURE 1:**
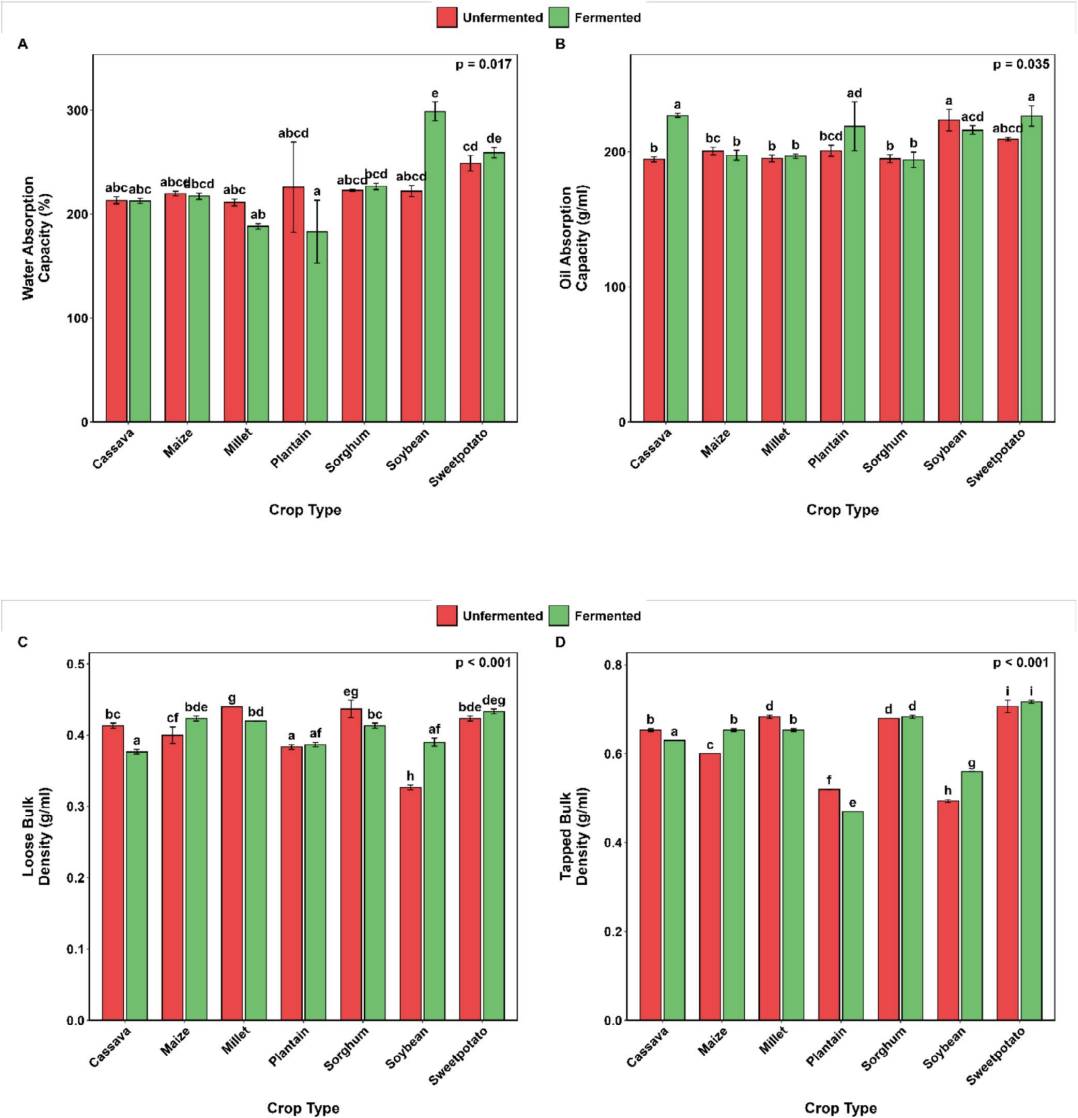
Changes in water (A) and oil (B) absorption capacities, loose (C) and tapped (D) bulk densities of selected Ghanaian plant‐based flour with SF. Bar values are means (*n* = 3). Error bars represent standard deviation. Means with different alphabets are significantly different (*p* < 0.05).

**FIGURE 2:**
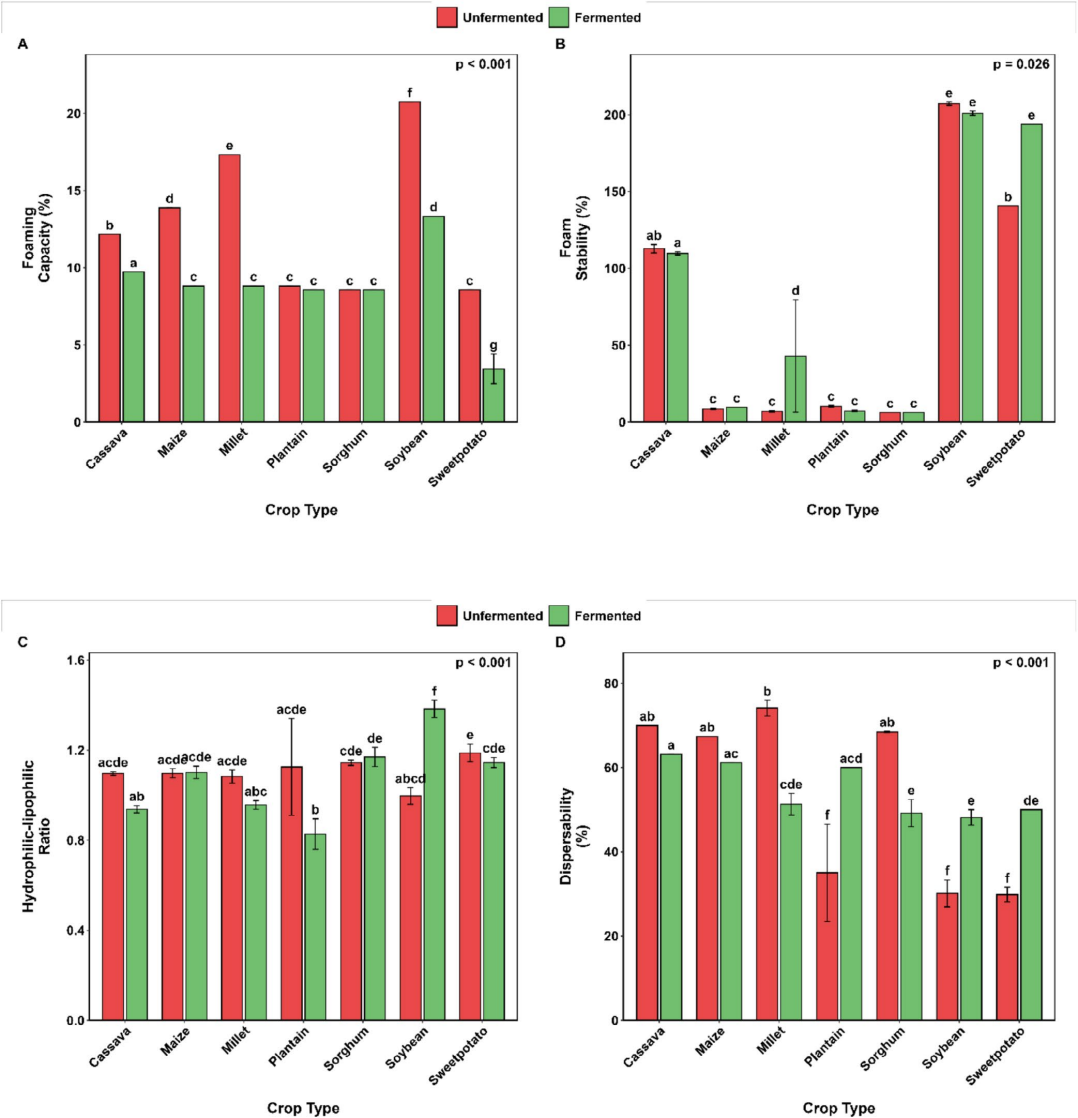
Changes in foaming capacity (A), foam stability (B), hydrophilic–lipophilic ratio (C), and dispersibility (D) of selected Ghanaian plant‐based flour with SF. Bar values are means (*n* = 3). Error bars represent standard deviation. Means with different alphabets are significantly different (*p* < 0.05).

**FIGURE 3:**
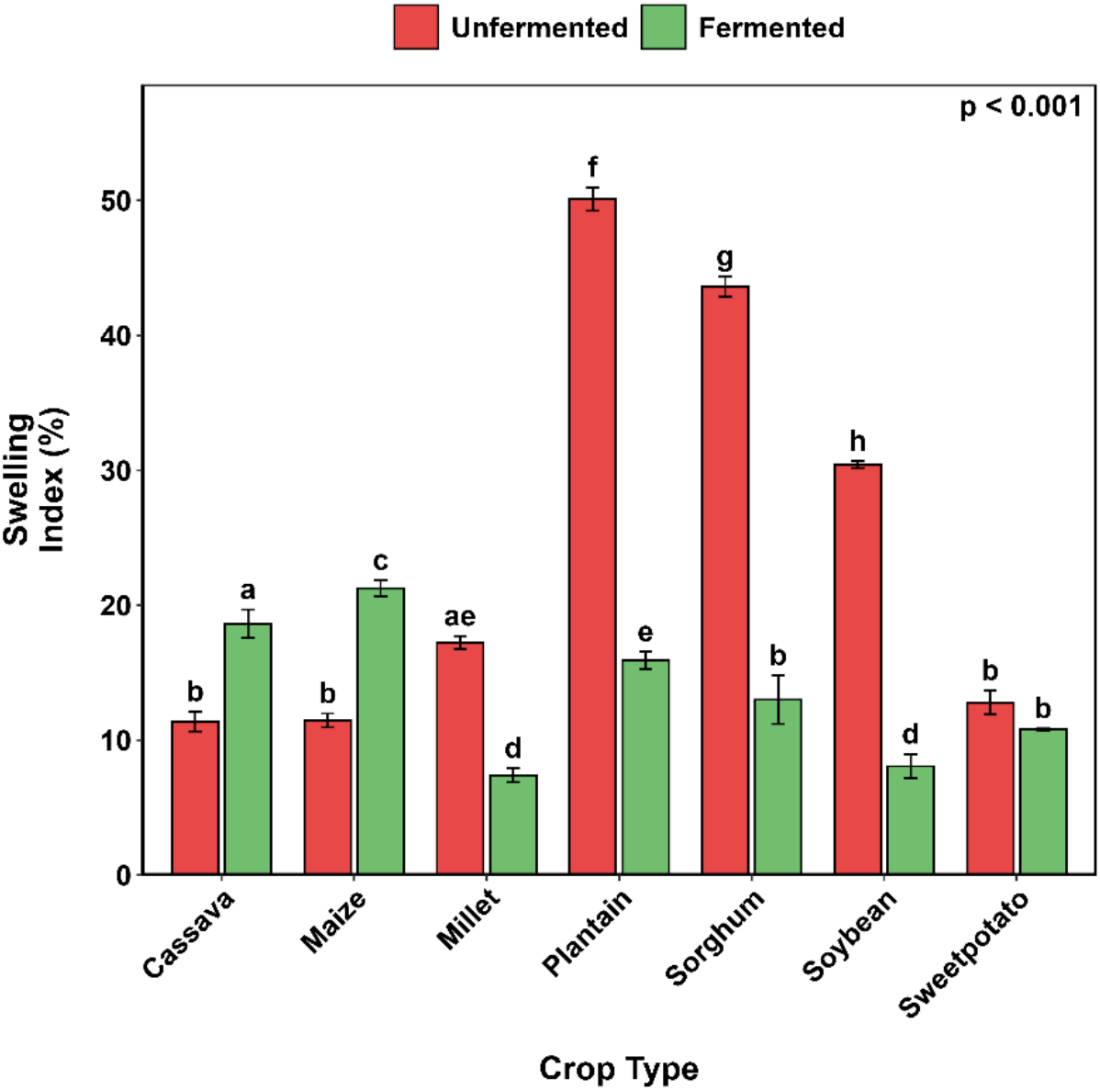
Changes in the swelling index of selected Ghanaian plant‐based flour SF. Bar values are means (*n* = 3). Error bars represent standard deviation. Means with different alphabets are significantly different (*p* < 0.05).

The functional properties of fermented flours from selected Ghanaian cereals, legumes, roots, tubers, and plantain are presented in Figures 1, 2, 3. SF did not considerably alter the water absorption capacity (WAC) of the flours, except for soybean flour, which exhibited notable changes (222.2 vs. 298.7%; *p* = 0.001) in water absorption (Figure 1A). A noteworthy increase in oil absorption capacity (OAC) was observed post‐fermentation (Figure 1B) in sweetpotato (209.4 vs. 226.5%), cassava (194.5 vs. 226.9%), and plantain (200.8 vs. 218.8%) flours.

Fermentation had different effects on the loose and tapped bulk densities of the flours investigated (Figure 1C,D). It is essential to note that significant changes occurred in both parameters, except that the loose bulk densities of cassava and millet flours were reduced by 8.7% and 4.6%, respectively. In contrast, the maize and soybean flours increased by 1.1‐fold and 1.2‐fold, respectively, for loose bulk density (Figure 1D). Overall, fermentation led to a considerable (*p* < 0.001) reduction in foaming capacity across all evaluated flours (Figure 2A). However, fermented millet and sweetpotato flours exhibited significantly greater foam stability (*p* = 0.03) than their unfermented counterparts (Figure 2B). SF affected the hydrophilic–lipophilic ratio (HLR) of the selected flour. In particular, it significantly reduced the HLR of plantain flour by approximately 1.4‐fold, but caused almost a 1.4‐fold increase in the HLR of soybean flour (Figure 2C).

SF significantly reduced the dispersibility of millet (74.1% vs. 51.3%) and sorghum (68.4% vs. 49.2%) flours. In contrast, there was a substantial (*p* < 0.001) increase in the HLR of plantain (35% vs 60%), soybean (30.2% vs 48.2%), and sweetpotato (29.8% vs 50.0%) flours (Figure 2D).

SF resulted in increases of 65.5% and 85.5% in the swelling index of cassava and maize flour, respectively. However, a substantial reduction in the swelling index was observed across all other flours investigated in this study (Figure 3).

### 3.2 Color

**FIGURE 4:**
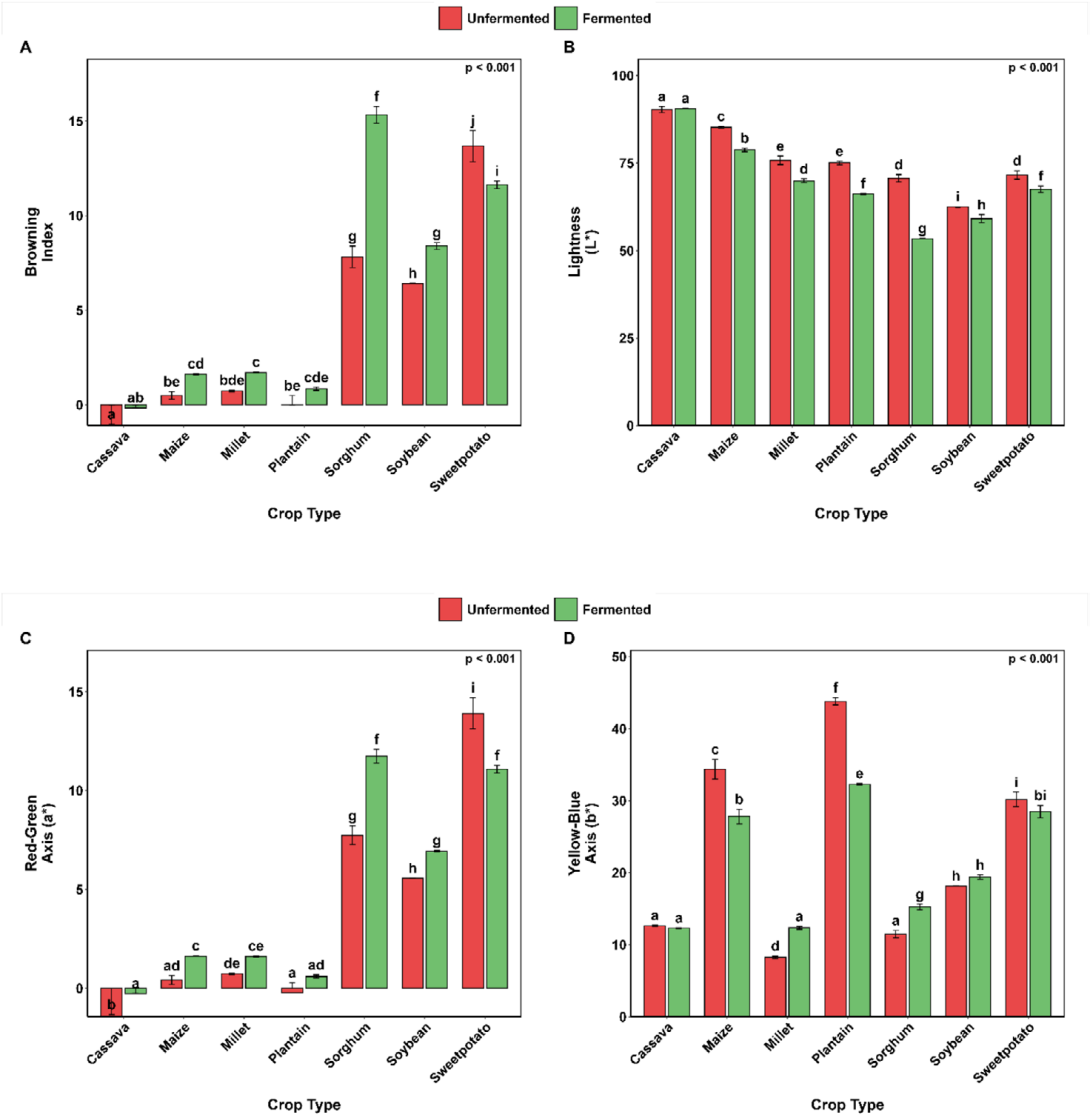
Color parameters (A–D) as influenced by SF and crop type. Bar values are means ± standard deviation (*n* = 3). Means with different alphabets are significantly different (*p* < 0.05).

The effect of SF on the color of different types of crops (cassava, maize, millet, plantain, sorghum, soybean, and sweetpotato) was assessed in terms of multiple color indices, as shown in Figure 4. The index of browning (BI) increased significantly (*p* = 0.001) after SF in most crops, although the increases were largest in sorghum and soybean (nearly 2‐ and 1.3‐fold, respectively) (Figure 4A). Conversely, sweetpotato SF significantly reduced BI by approximately 15%. Cassava had the lowest BI, and fermentation had no significant effect on it. Similar trends were observed for maize, millet, and plantain, whose fermentation processes resulted in a statistically insignificant increase in BI.

Lightness (L*) varied significantly (*p* = 0.001) among crops following SF, with cassava having the highest lightness and soybean the lowest (Figure 4B). Although the effect of SF was not significant on cassava samples, it produced a substantial decrease in lightness in all other crop samples.

The red‐green chromaticity (a*) of the soybean and sorghum samples increased after SF, whereas that of sweetpotato decreased (Figure 4C). The redness increased by 1.5‐ and 1.3‐times in the sorghum samples, respectively, but sweet potato samples were found to be reduced in redness by 1.3 times.

SF significantly influenced the yellowness (b*) of all crops (*p* < 0.001), with plantain and maize exhibiting the highest values (Figure 4D). It significantly (*p* < 0.001) decreased the b* values in maize, plantain, and sweetpotato by approximately 1.2, 1.4, and 1.1 times, respectively. Millet and sorghum, on the other hand, demonstrated a considerable rise in b* value distinguishing them after fermentation, by 8.3 to 12.4 and 11.5 to 15.3, respectively (*p* < 0.001).

**FIGURE 5:**
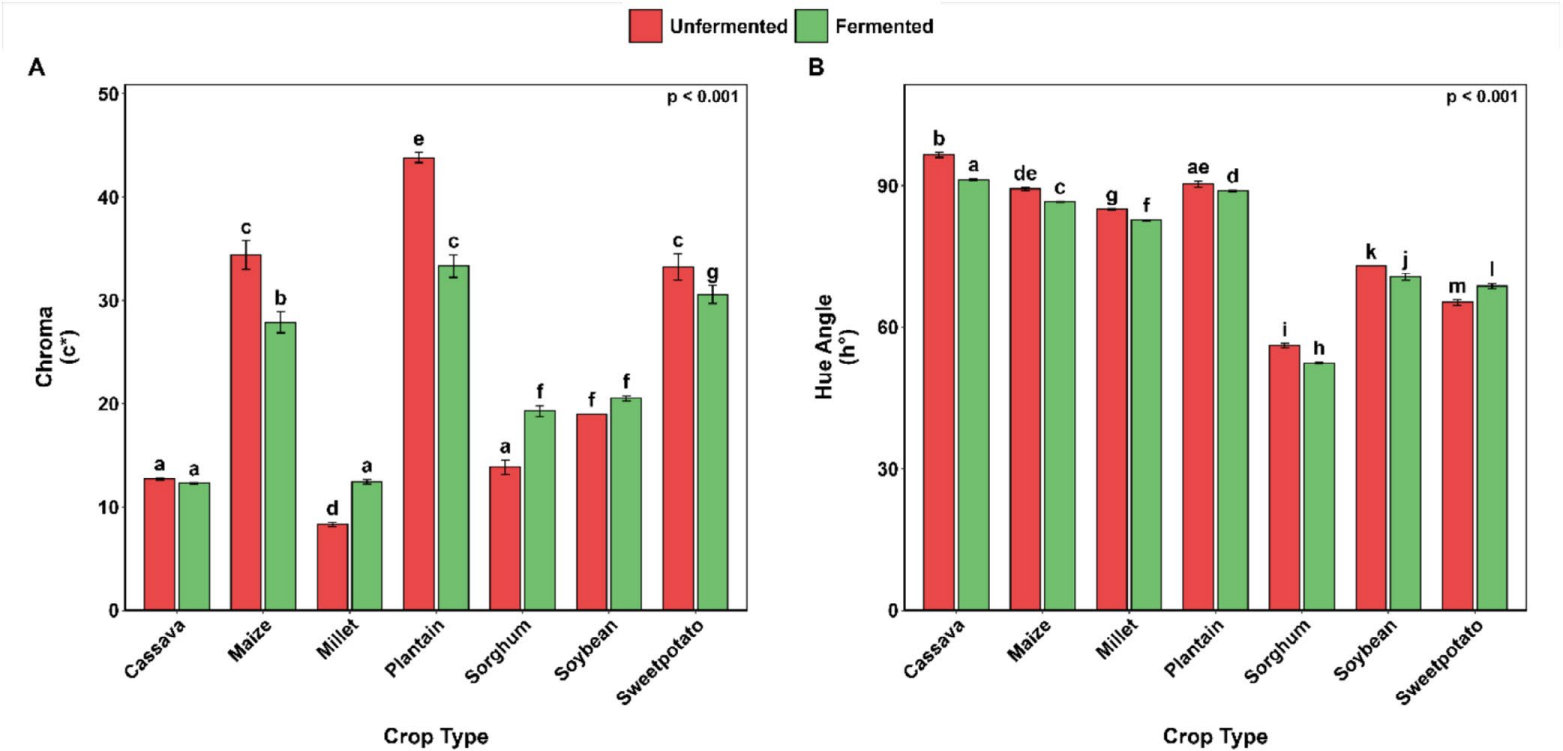
Chroma (A) and Hue angle (B) as influenced by SF and crop type. Bar values are means ± standard deviation (*n* = 3). Means with different alphabets are significantly different (*p* < 0.05).

A similar effect was observed for chroma (C), where SF induced significant decreases in maize, plantain, and sweetpotato by approximately 6.5, 10.5, and 2.6 units, respectively (*p* < 0.001; Figure 5A). In contrast, there was an extreme increase in the chroma values of millet and sorghum after fermentation (*p* < 0.001). No significant alterations in chroma were observed in the case of soybean or cassava.

SF had a significant influence on the hue angle (h °) of all crops (*p* < 0.001), with cassava showing the highest h ° and sorghum the lowest (Figure 5B). Chroma values after the fermentation process decreased the most in cassava flour, by 5.2 units. Plantain flour had the smallest reduction, 0.4 units.

**FIGURE 6:**
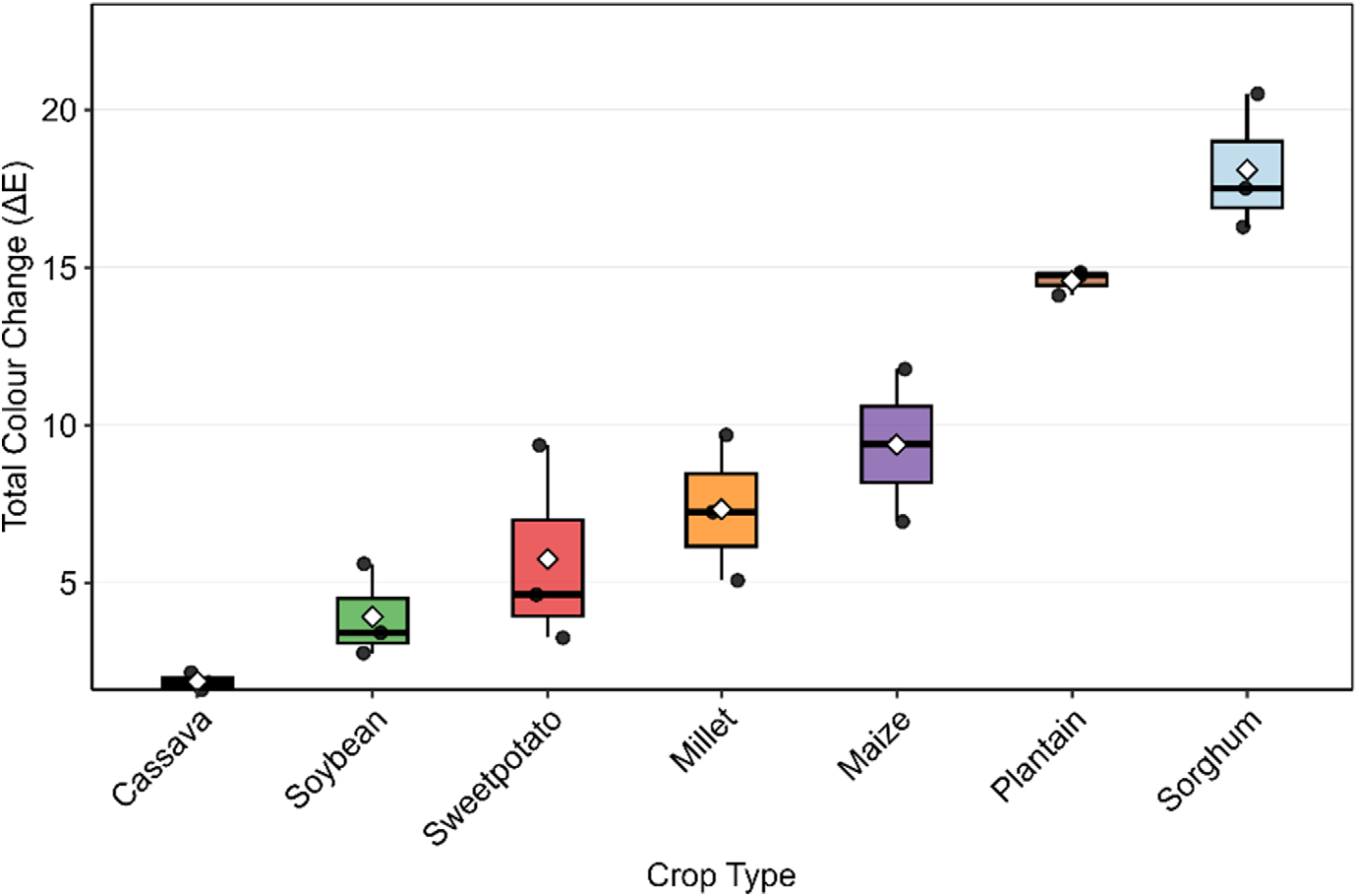
Total color change after open‐air drying of fermented Ghanaian flours. Values are means ± standard deviation (*n* = 3).

Sorghum flour displayed the most pronounced color alteration among the tested crops after SF, with the highest median ΔE. In contrast, cassava flour exhibited the least (Figure 6). Maize and millet flours showed moderate color changes, with no significant difference between them following SF. Sweetpotato and soybean flours showed significantly lower color changes than maize flour, whereas cassava flour showed minimal alterations.

### 3.3 Pasting Properties

The effect of SF on pasting parameters varied by crop type, as shown in Table 1. In general, peak viscosity increased significantly (*p* < 0.001) in most flours following fermentation, except for cassava and sorghum flours, which exhibited a decrease of approximately 14% and 18%, respectively. Trough viscosity, also referred to as hot paste viscosity, showed a significant increase (*p* < 0.001) across all fermented flours, except for sorghum flour, where a decline was observed (Table 1).

**TABLE 1:** Effect of SF on selected indigenous Ghanaian cereals, legumes, roots, tubers and plantain flours.

Crop	Process	Pasting properties						
		Peak viscosity (cP)	Trough (cP)	Breakdown (cP)	Final viscosity (cP)	Setback (cP)	Peak time (min)	Pasting temp (°C)
Cassava	Unfermented	5344.50 ± 48.8^b^	2540.00 ± 9.9^b^	2804.50 ± 58.7^b^	3187.50 ± 12.0^e^	647.50 ± 2.1^f^	4.50 ± 0.0^g^	73.50 ± 0.1^f^
	Fermented	4622.50 ± 17.7^c^	2636.00 ± 21.2^b^	1986.50 ± 38.9^c^	3597.50 ± 16.3^d^	961.50 ± 5.0^def^	4.87 ± 0.2^f^	74.32 ± 0.0^f^
Maize	Unfermented	1200.00 ± 70.7^i^	952.00 ± 12.7^g^	189.00 ± 0.0^gh^	2068.00 ± 2.8^fg^	1114.00 ± 7.1^de^	5.45 ± 0.1^de^	87.95 ± 0.0^ab^
	Fermented	1675.00 ± 2.8^h^	1239.50 ± 2.1^f^	440.50 ± 7.8^fg^	2367.00 ± 8.5^f^	1128.00 ± 7.1^de^	5.91 ± 0.0^b^	90.767 ± 0.6^a^
Millet	Unfermented	1959.00 ± 403.1^g^	1424.00 ± 212.1^e^	535.00 ± 190.9^ef^	2241.50 ± 27.6^f^	817.50 ± 184.6^ef^	5.77 ± 0.3^bc^	88.03 ± 5.8^ab^
	Fermented	2251.50 ± 16.3^f^	1521.00 ± 29.7^e^	730.50 ± 13.4^e^	2260.00 ± 8.5^f^	734.50 ± 14.9^f^	5.60 ± 0.0^cd^	83.60 ± 0.6^cd^
Plantain	Unfermented	2215.50 ± 119.5^f^	1460.50 ± 67.2^e^	2138.50 ± 128.0^c^	6780.00 ± 608.1^a^	7550.00 ± 523.3^a^	5.33 ± 0.0^e^	86.50 ± 0.1^bc^
	Fermented	6170.00 ± 14.1^a^	6095.00 ± 7.1^a^	7700.00 ± 367.7^a^	1855.00 ± 7.1^g^	7.50 ± 0.7^g^	6.44 ± 0.1^a^	88.00 ± 0.1^ab^
Sorghum	Unfermented	3243.00 ± 5.7^d^	2215.50 ± 37.5^c^	1027.50 ± 31.8^d^	5450.50 ± 41.7^b^	3235.00 ± 79.2^b^	5.33 ± 0.0^e^	81.03 ± 0.6^de^
	Fermented	2650.50 ± 24.8^e^	2056.00 ± 33.9^d^	594.50 ± 9.2^ef^	4330.00 ± 43.8^c^	2274.00 ± 9.9^c^	5.40 ± 0.1^de^	84.73 ± 0.0^bc^
Soybean	Unfermented	23.00 ± 0.0^j^	18.00 ± 1.4^i^	26.50 ± 0.7^h^	9.00 ± 0.0^i^	6.50 ± 0.7^g^	1.08 ± 0.0^j^	58.50 ± 0.7^g^
	Fermented	22.00 ± 0.0^j^	19.50 ± 0.7^i^	19.50 ± 0.7^h^	2.00 ± 1.4^i^	3.50 ± 0.7^g^	1.65 ± 0.1^i^	55.00 ± 0.0^g^
Sweetpotato	Unfermented	1139.50 ± 7.8^i^	581.50 ± 0.7^h^	558.00 ± 8.5^ef^	865.50 ± 2.1^h^	284.00 ± 1.4^g^	4.20 ± 0.1^h^	79.55 ± 0.5^e^
	Fermented	3161.50 ± 10.6^d^	1928.00 ± 1.4^e^	1233.50 ± 9.2^d^	3099.00 ± 22.6^e^	1171.00 ± 24.0^d^	5.00 ± 0.1^f^	80.33 ± 0.7^de^
*p*‐value		< 0.001	< 0.001	< 0.001	< 0.001	< 0.001	< 0.001	0.020

*Note:* Values are means ± standard deviation, *n* = 3. Values in the same column sharing different letters are significantly different (*p* < 0.05).

All flour samples, except those of plantain and sweetpotato, showed decreases in breakdown viscosity, with exceptionally high increases of approximately 3.6 and 2.2 fold, respectively, in plantain and sweetpotato flours (Table 1). Most of the tested flours exhibited a significant increase in final viscosity after SF. Nevertheless, plantain and sorghum flours did not follow this pattern, as they recorded decreases of approximately 72.6% and 20.6%, respectively, after the SF treatment. Fermentation of cassava, maize, and sweetpotato flours also resulted in a significant increase in setback viscosity (*p* < 0.001). In contrast, millet, sorghum, and plantain flours showed varying levels of reduction (Table 1). All fermented flours, except millet, had a significant (*p* < 0.001) increase in peak time, which is the time required to achieve maximum viscosity. The same trend was observed for the pasting temperature, which was slightly lower in fermented millet flour (by 5%) than in the unfermented flour (Table 1).

### 3.4 Compositional Profile

**FIGURE 7:**
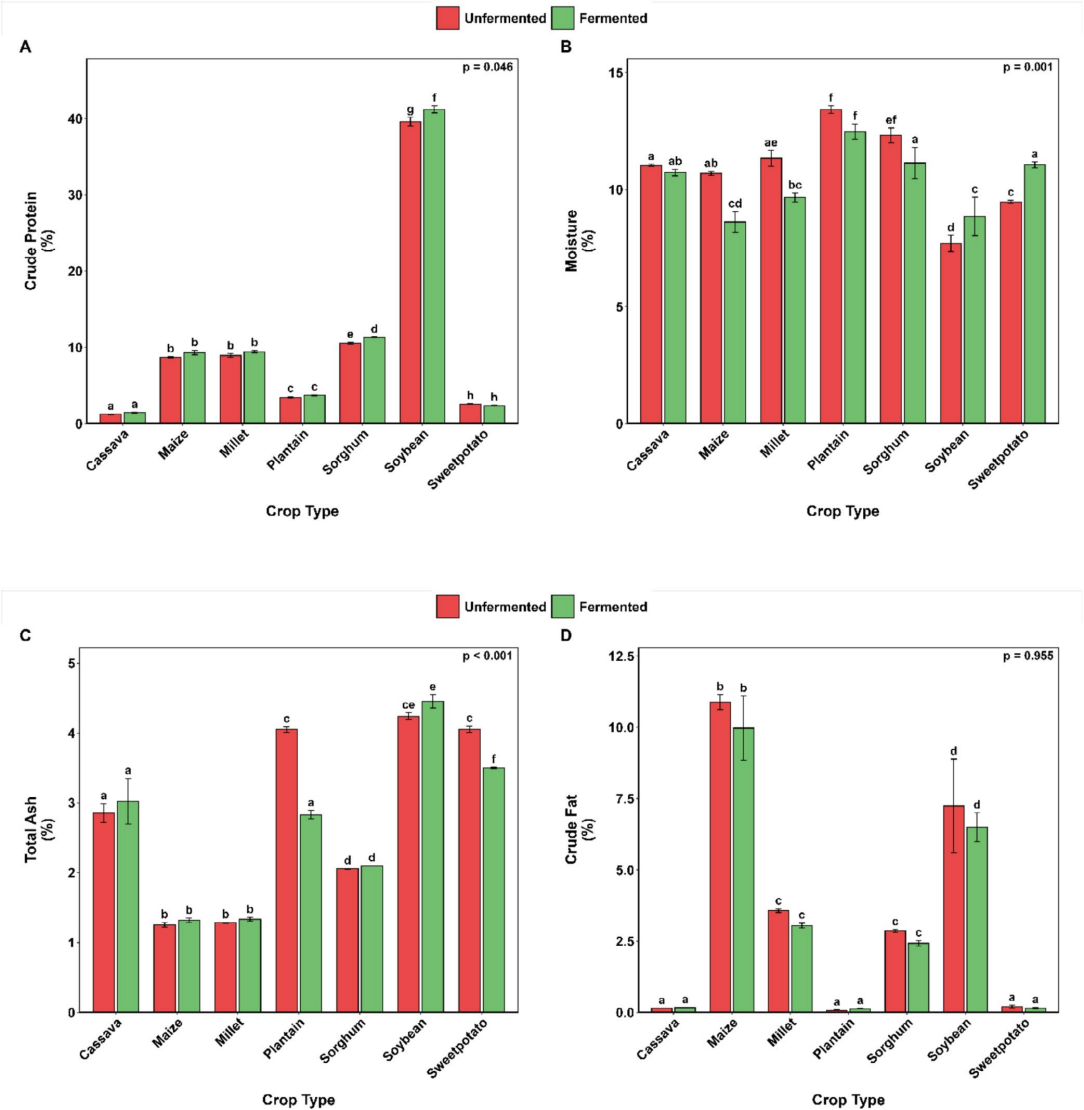
Changes in crude protein (A), moisture (B), total ash (C), and crude fat (D) content of selected Ghanaian plant‐based flour with SF. Bar values are means ± standard deviation (*n* = 2) expressed on a dry matter basis except for moisture (B). Means with different alphabets are significantly different (*p* < 0.05).

**FIGURE 8:**
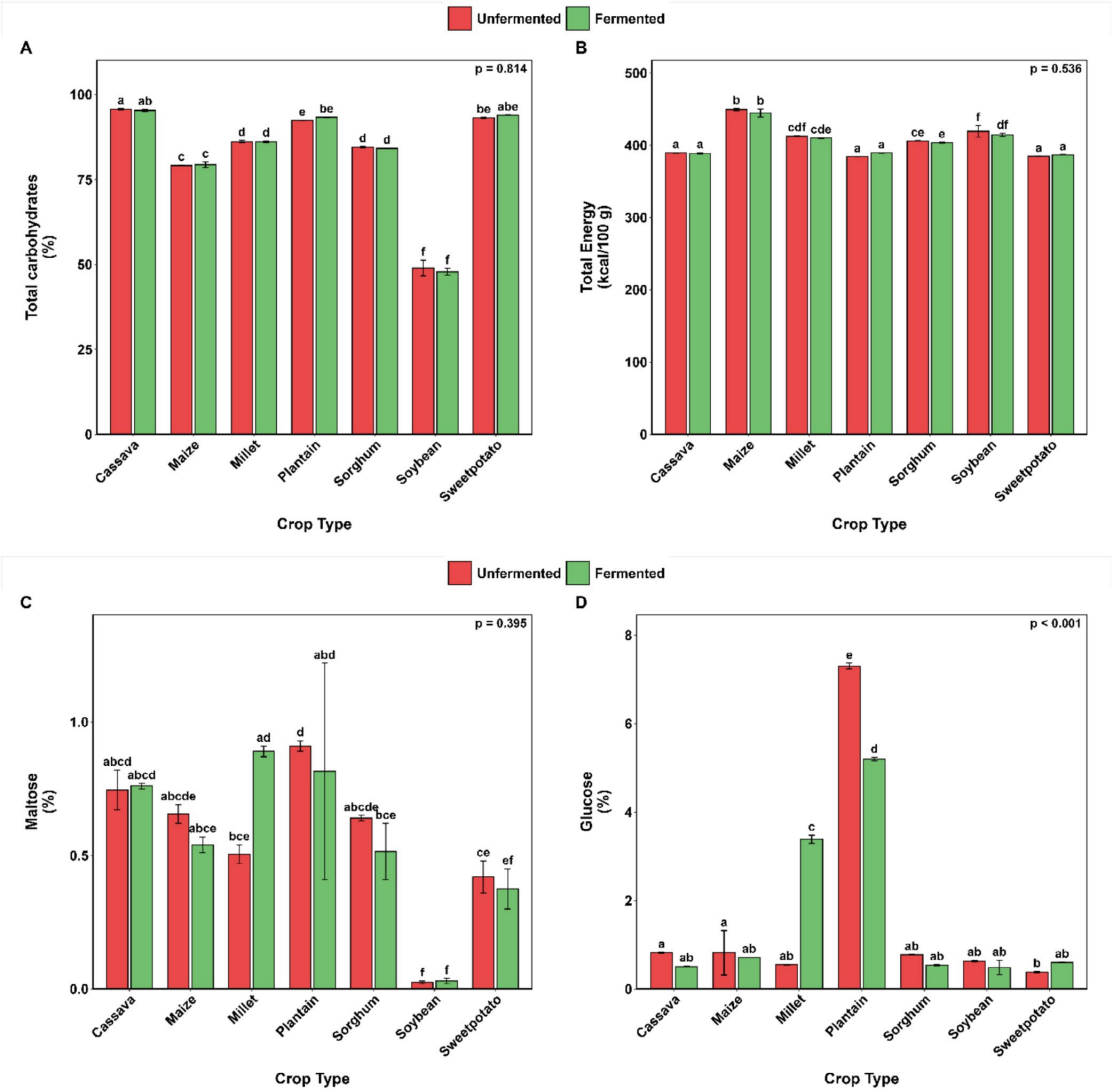
Changes in total carbohydrates (A), energy (B), maltose (C), and glucose (D) content of selected Ghanaian plant‐based flour with SF. Bar values are means ± standard deviation (*n* = 2) expressed on a dry matter basis. Means with different alphabets are significantly different (*p* < 0.05).

Figures 7 and 8 illustrate the impact of SF on the proximate composition, total energy content, and simple sugar profiles of selected cereals, legumes, roots, tubers, and plantains. As anticipated, the crops exhibited significant variation in crude protein content, with soybean displaying the highest levels and cassava the lowest (Figure 7A).

Notably, among all crops, only soybean showed a significant increase in crude protein due to SF, while the others showed only marginal differences. The sweetpotato flour showed a marginal decrease in its crude protein concentration after SF (Figure 7A).

All samples had relatively low moisture contents (< 15%). SF mediated the moisture content of the samples in different ways. There was a considerable drop in moisture levels for maize, millet, sorghum, and plantain, whereas soybeans and sweetpotato showed an increase in moisture levels after the SF (Figure 7B).

The total ash composition characterized the total mineral content of the crops, with soybeans having the highest, followed by maize and millet (Figure 7C). Notably, only plantain and sweetpotato showed a significant reduction in total ash content following SF treatment.

Although the effect of SF on the crude fat content was insignificant (*p* = 0.955) for all the flours investigated, it generally resulted in marginal declines in most flours. The notable decreases were observed in maize (8.5%), millet (14.6%), sorghum (15.2%), and soybean (10.2%) flours (Figure 7D). The starchy crops (sweetpotato, cassava, and plantain) exhibited the highest total carbohydrate content (92.4%–95.8%), whereas soybean (a legume) showed the lowest values, ranging from 39.7% to 43.4% (Figure 8A). Cereals (maize, millet, and sorghum) demonstrated intermediate carbohydrate levels (79.1%–86.2%). SF generally did not significantly affect carbohydrate content, except in soybeans, where it increased it by almost 9.4%.

SF did not substantially alter the calorific values of most crops, except for soybeans, which showed a 6.2% decrease in value after fermentation. In contrast, the plantain matrix (Figure 8B) showed a different response, with a 1.3% increase in the calorific value after fermentation.

**FIGURE 9:**
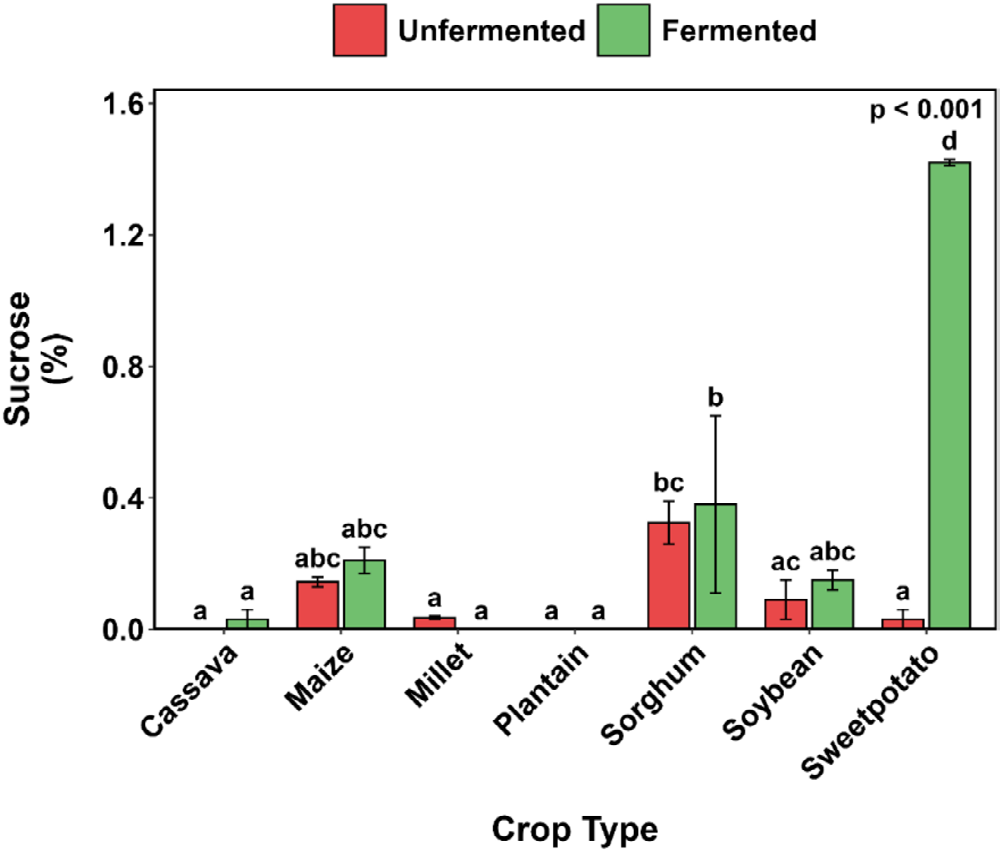
Changes in sucrose content of selected Ghanaian plant‐based flour with SF. Bar values are means ± standard deviation (*n* = 2) expressed on a dry matter basis. Means with different alphabets are significantly different (*p* < 0.05).

Maltose levels remained largely unaffected by SF across all crops (Figure 8C), with only millet showing a marked increase of approximately 76%. Glucose levels showed crop‐specific responses: plantain exhibited significantly higher initial glucose content that decreased post‐fermentation, whereas millet showed the opposite trend (Figure 8D). Other crops did not show significant reductions in glucose content after SF. Sucrose concentrations were generally low across all substrates; however, SF resulted in an exponential increase in sucrose content (Figure 9).

**FIGURE 10:**
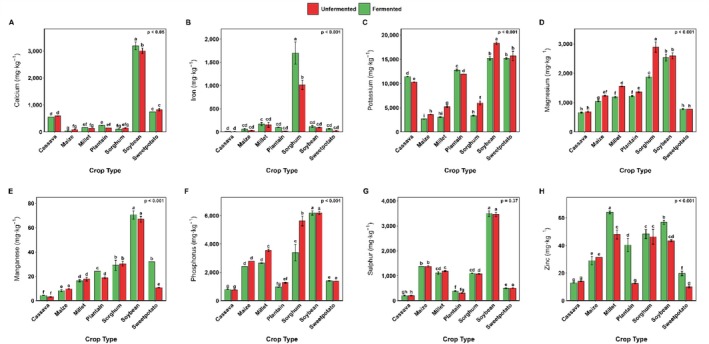
The effect of SF on the mineral composition of selected Ghanaian cereals, legumes, root, tubers, and plantain flour. Values are means ± standard deviation (*n* = 2) expressed on a dry matter basis.

As expected, the various crops differed in their mineral content, with soybeans showing particularly high levels of calcium, potassium, phosphorus, manganese, and sulfur (Figure 10). The specific influence of SF on the selected food matrices also varied significantly with crop type. For instance, whereas SF caused a marginal decrease in the calcium content in cassava, maize, sorghum, and sweetpotato flours, the reverse was observed in the flours of millet, plantain, and soybean (Figure 10A). A similar trend was observed for zinc, except for cassava and maize flour, which exhibited marginal declines (Figure 10H). In the case of iron, a stable to marginal increase was observed across all the selected crops. The increase in iron content was prominent in sorghum flour after fermentation (Figure 10B). Apart from cassava and plantain flours, fermentation significantly decreased the potassium content in the flours investigated. Specifically, the reductions were approximately 26% in maize, 41% in millet, 44% in sorghum, 17% in soybean, and 3.4% in sweetpotato flours (Figure 10C). Generally, magnesium and phosphorus contents decreased across the substrates following fermentation.

### 3.5 Total Phenolic Content

**FIGURE 11:**
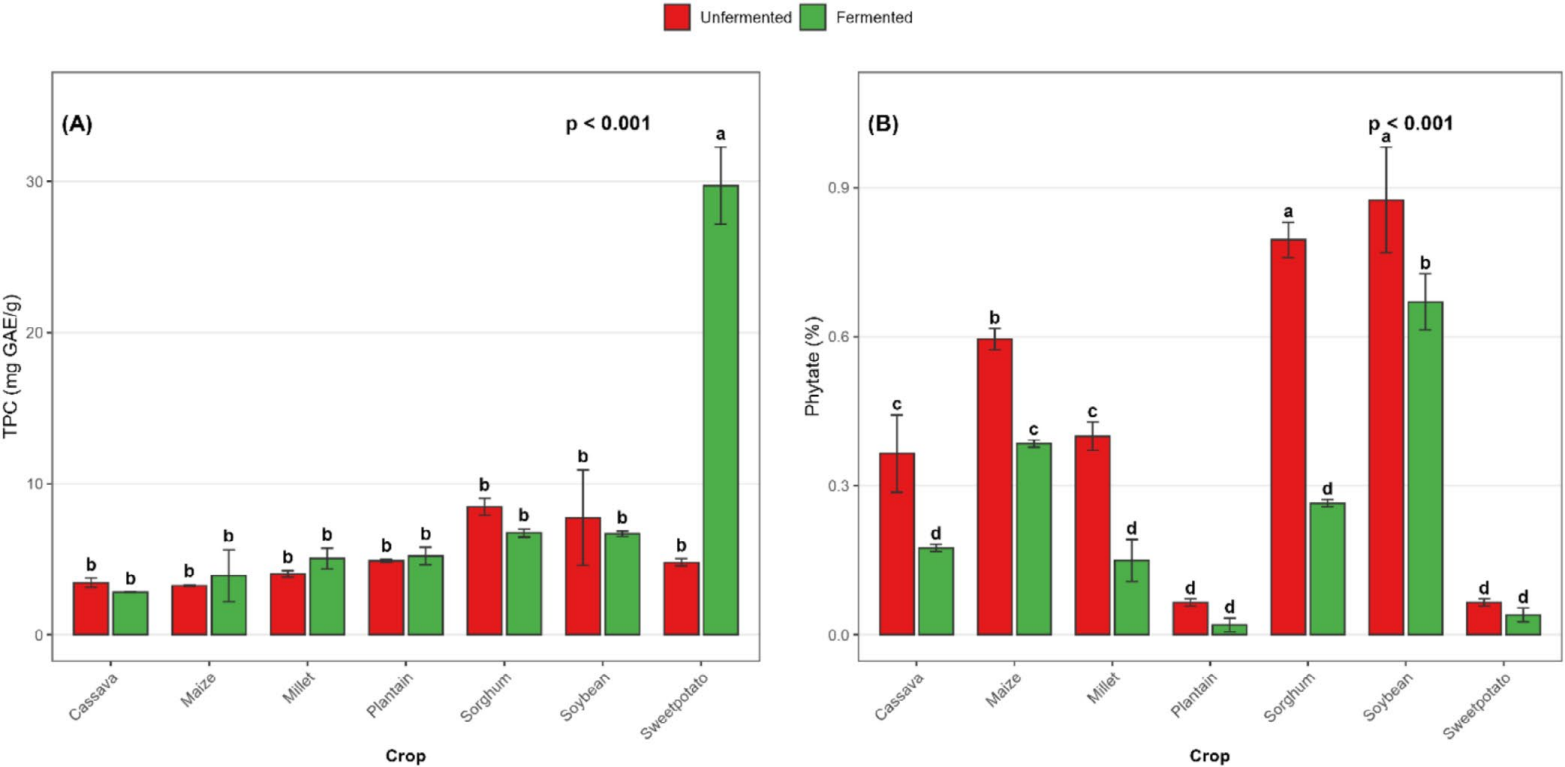
Effect of SF on the total polyphenolic (A) and phytic acid (B) content of selected Ghanaian cereals, legumes, root, tuber, and plantain flours. Bar values are means ± standard deviation (*n* = 2) expressed on a dry matter basis. Means with different alphabets are significantly different (*p* < 0.05).

Apart from sweetpotato flour, SF did not significantly affect the total phenolic content across all crop matrices investigated. Specifically, SF increased the total phenolic content of sweetpotato flour by 6.2 fold (Figure 11A).

### 3.6 Phytic Acid

The phytic acid content generally decreased significantly (*p* < 0.001) following SF, except for plantain and sweetpotato, where the reduction was insignificant. Specifically, the reduction was 52.1% in cassava, 35.3% in maize, 62.5% in millet, 69.2% in plantain, 66.7% in sorghum, 23.4% in soybean, and 38.5% in sweetpotato flour (Figure 11B). The data further showed that the phytic acid content was generally higher (0.15 g–0.85 g/100 g) in the grains and legumes than in the starchy roots, tubers, and plantain substrates (0.02 g–0.37 g/100 g).

## 4 Discussion

SF slightly reduced the WAC, indicating the optimal amount of water to add to a dough before it becomes excessively sticky in most flours analyzed, except for soybean and sweetpotato flours. These disparities underscore the intricate nature of food matrix interactions under conventional processing conditions. In a previous study, Atuna et al. (2021) noted that the effects of processing techniques, including fermentation, are highly matrix‐specific; that is, the structural and compositional properties of each flour type are essential determinants of the outcome. The significant increase in the WAC of both soybean and sweetpotato flours during the observed period could be attributed to protein hydrolysis during SF, which exposed or formed more polar amino acid residues. These residues also increase the water‐binding capacity of flour, as reported by Ntso et al. (2016). This suggests that, in specific matrices, SF may improve water absorption rather than diminish it, as reported for maize‐based products by Afoakwa et al. (2007).

The increase in OAC among the starchy root, tuber, and plantain flours corroborates the findings of Wang et al. (2020) who reported increased oil absorption with fermentation in potato flour. The observed increase in the OAC of these flours could be attributed to the denaturation and detachment of protein components, which exposed the nonpolar side of the protein molecules (Awuchi et al. 2019). OAC is a key functional property that enhances mouthfeel and helps retain the flavor of food products (Adebowale and Lawal 2004).

Among the flours investigated, only soybean showed a significant increase in the hydrophilic–lipophilic ratio, likely due to the biochemical changes during fermentation. HLR reflects the balance between polar and nonpolar functional groups within food materials and influences their behavior in aqueous and lipid systems. For instance, some fermentation processes may alter or reduce lipid structures, thereby decreasing the lipophilic characteristics of flour.

Bulk density is a vital attribute of flour products and a significant factor in package design, material treatment, and use in food items (Gong et al. 2020). The present decrease in bulk density after fermenting cassava and millet flours is consistent with other reports of decreased bulk density after flour fermentation (Adedeji et al. 2015; Elkhalifa et al. 2005; Sreerama et al. 2009). The reduction in bulk density following SF treatment indicates that a higher flour concentration is required to prepare a thinner porridge, thereby increasing its energy density (Ojokoh et al. 2015). However, the increase in bulk density in fermented maize and soybean could be attributed to the partial retrogradation of starch (Wu et al. 2022), which may have resulted in more compact crystalline structures, thereby increasing the density.

Dispersibility, which is the ability of flours to reconstitute effectively by absorbing water without lump formation, is accompanied by the rapid disintegration of agglomerated particles (Otegbayo et al. 2013; Tanimola et al. 2016). In this study, SF had different effects on flour dispersibility depending on its source. It reduced dispersibility in cereal and cassava, likely due to the tight molecular structure; however, it improved dispersibility in plantain, soybean, and sweetpotato, possibly due to enzymatic degradation of starch and associated non‐starch polysaccharides, with partial protein hydrolysis further enhancing dispersibility (Chaves‐López et al. 2020). These opposing effects highlight the importance of planning fermentation to achieve the desired functional properties for specific food applications in the future. The observed considerable reduction in cereal flour dispersibility following SF is consistent with the findings of Oloyede et al. (2016), who reported a similar trend in defatted moringa seed flour.

The reduction in foaming capacity observed following SF in this study supports the findings of Chikelu et al. (2025) who also reported a decrease in the foaming capacity of fermented cocoyam flour. This decline is commonly attributed to the structural and conformational changes in proteins that occur during fermentation. Foaming capacity is highly dependent on protein configuration, flexibility, and surface activity; any alteration in the native structure can significantly impair the ability to form and stabilize foam. This finding supports that of Xiao et al. (2018) who reported an almost 9% increase in foam stability following fermentation of red beans. The increased foam stability has been attributed to structural changes in the protein during solid‐state fermentation, which tend to enhance intermolecular interactions, thus improving the viscoelastic film formed (Xiao et al. 2018).

The observed color transformations during SF reflect the complex interactions between crop‐specific phytochemical profiles and microbial metabolic activity. The differential browning responses across crops align with established patterns of phenolic compound reactivity, where substrates with higher polyphenol content (e.g., sorghum, soybean, and sweetpotato) typically exhibit more pronounced Maillard and enzymatic browning (Manzocco et al. 2000). As previously reported, the exceptional behavior of sweetpotato, showing reduced browning, could be attributed to the synthesis of metabolites with strong antioxidant activity by LAB ( *L. acidophilus* CH‐2) and the degradation of browning products during fermentation (Bell et al. 2016; Li et al. 2021). The drop in pH during fermentation could also have negatively affected *polyphenol oxidase* (PPO), an enzyme responsible for enzymatic browning in polyphenol‐rich materials. The optimum pH for PPO activity is approximately 6.5–7.0 (Liu et al. 2015).

Lightness variations demonstrate fundamental differences in substrate‐microbe interactions. The general darkening trend corresponds with previous reports of pigment polymerization and melanoidin formation during fermentation (Dulf et al. 2016), while cassava's stability supports the hypothesis that high‐starch, low‐phenolic matrices resist color modification (Champagne et al. 2011). The crop‐specific responses in chromaticity parameters (a* and b*) reveal distinct pigment transformation pathways, where microbial communities appear to either degrade native pigments (as in carotenoid‐rich crops) or synthesize new chromophores (particularly in phenolic‐rich cereals). These findings extend current understanding of fermentation‐induced color chemistry by demonstrating how substrate composition directs pigment metabolism pathways.

The observed chroma and hue angle patterns provide new insights into the dynamics of fermentation‐induced color. The reduced color intensity in certain crops suggests either pigment dilution or the formation of larger molecular weight compounds through oxidative coupling (Olukomaiya et al. 2020) whereas increased chroma in others indicates microbial production of stable chromophores. The hue angle shifted toward redder tones in most crops, corroborating previous findings on fermented plant matrices (Pandiselvam et al. 2023). However, the persistent yellowness of cassava presents an interesting exception that may be related to its unique cyanogenic glucoside content (McMahon et al. 1995).

The significant total color differences (ΔE) across crops highlight the importance of matrix effects on fermentation outcomes. However, the unexpected stability of some pigmented crops suggests alternative stabilization mechanisms, possibly through microbial production of antioxidants or structural protection by starch granules (Yuliana et al. 2025). These findings have important implications for product development, suggesting that fermentation protocols may need to be optimized based on both desired nutritional outcomes and color preservation requirements.

### 4.1 Pasting Properties

Fermentation substantially affects the pasting properties of the selected indigenous crops, with direct implications for their suitability as complementary food ingredients. Peak viscosity increased in most flours following fermentation, likely due to enhanced starch swelling, a common phenomenon during fermentation. This may not be desirable for infant feeding, as excessively viscous gruels can limit the intake and reduce the energy density (Amagloh et al. 2013). However, the reduced peak viscosity observed in fermented cassava and sorghum flours may be advantageous, enabling thinner, more easily swallowable preparations. Peak viscosity is the maximum viscosity during cooking or heating and is typically correlated with the final product quality (Atuna et al. 2021).

The observed increase in trough viscosity (hot paste viscosity) in most flours following fermentation suggests enhanced thermal stability, which is beneficial for maintaining texture and consistency during reheating processes. This improvement likely reflects structural changes in starch granules or the formation of fermentation‐derived metabolites that stabilize the paste matrix under heat. Such thermal resilience is advantageous for food safety and consumer convenience, as it enables reheating without significant loss of quality. Conversely, the decrease in trough viscosity observed in sorghum flour may indicate reduced ability to retain viscosity under heat, potentially leading to altered cooking behavior and a less desirable texture in final products. This decline could result from enzymatic degradation of starch or other macromolecules during fermentation, which weakens the paste structure. The differential response between sorghum and other flours underscores the substrate‐specific effects of fermentation on rheological properties, emphasizing the need to tailor fermentation conditions to preserve or enhance functional qualities depending on the crop.

The breakdown viscosity generally decreased post‐fermentation, reflecting greater shear resistance, which is beneficial for processing and mixing. Nevertheless, the sharp increases in plantain and sweetpotato flour viscosities suggest fragility under heat, potentially leading to undesirable thickening as complementary food ingredients. Amagloh (2022) identified sweetpotato as suitable for complementary foods because its porridges exhibit lower viscosity than cereal‐based blends. A limitation of sweetpotato, despite its initial suitability (Amagloh 2022), is that SF may increase its viscosity to levels that are less ideal for infant feeding. Future research should explore optimized SF protocols or blending strategies to mitigate this increase while retaining the other benefits.

The final viscosity trends further highlight this concern; while increased final viscosity may indicate stronger gel formation, it can also result in overly thick textures that are unsuitable for infants' consumption. The marked reductions in particle sizes of plantain and sorghum flours suggest improved post‐cooking fluidity.

Setback viscosity, associated with retrogradation, increased in cassava, maize, and sweetpotato flours, which may be less susceptible to retrogradation during cooling than other flours. The higher the setback value, the lower the retrogradation during cooling and the lower the staling rate of the products made from flour (Falade and Okafor 2015). In contrast, millet, plantain, and sorghum flours exhibited reduced setback values, indicating their susceptibility to retrogradation during cooling.

The observed increases in peak time and pasting temperature (except for millet) imply delayed gelatinisation and higher cooking energy requirements, which may constrain low‐resource settings. Overall, millet, sorghum, and plantain flours, despite some limitations, exhibited pasting profiles that were more aligned with the textural and digestibility requirements of complementary foods. In contrast, cassava, maize, and sweetpotato flours may require further blending to achieve an optimal feeding consistency.

### 4.2 Compositional Properties

The variations observed in the proximate composition among crops align well with the established botanical classifications, in which legumes, such as soybeans, typically accumulate higher levels of protein and minerals than traditional starchy staples (Vollmann 2016).

#### 4.2.1 Crude Protein

The significant protein enhancement in fermented soybeans is corroborated by previous findings on the mixed culture fermentation of soymeal (*Bacillus subtilis, Aspergillus niger*, *Saccharomyces cerevisiae* , and *Lactiplantibacillus plantarum*) (Sukhikh et al. 2022). The increased crude protein content could be attributed to the microbial proteolytic activity during legume fermentation (Senanayake et al. 2023). The insignificant change in crude protein content in most food substrates is consistent with the findings of Khetarpaul and Chauhan (1989), who reported that SF resulted in a negligible change in the protein content of pearl millet flour. The stability of protein content in other crops may suggest either limited proteolysis or simultaneous degradation and synthesis of crude proteins.

#### 4.2.2 Moisture Content

The moisture content of the flours has essential implications for product stability, as the achieved levels (< 15%) fall within the range known to inhibit most spoilage microorganisms (Atuna et al. 2021). The increase in soybean and sweetpotato may be explained by their higher initial soluble solid content, which facilitates osmotic water retention (Heo et al. 2021).

#### 4.2.3 Total Ash

The total mineral pattern reinforces the nutritional superiority of legumes, with the high total ash content of soybeans being consistent with their reported mineral density (Moisa et al. 2024). Consistent with the current study, the total ash content increased following the fermentation of mung bean flour (Onwurafor et al. 2014) and tamarind seeds (Olagunju et al. 2018). The higher ash levels reported in these studies resulted from microbial metabolic processes and the degradation of complex chelated substances in the fermenting mass, promoting increased mineral availability (Adebo et al. 2022). However, reports on reduced total ash following fermentation in *Phaseolus vulgaris* (Granito et al. 2002, 2005). The decrease in the ash content of plantain and sweetpotato is consistent with the findings of Falola et al. (2015) who observed a similar reduction in the total ash content of plantain flour following SF.

#### 4.2.4 Crude Fat

The SF‐induced changes in the lipid profiles merit particular attention. The general decline in crude fat content post‐fermentation aligns with earlier reports on maize (Ejigui et al. 2005; Ogodo et al. 2017), pearl millet (Adebiyi et al. 2016), sorghum (Mohammed et al. 2011), and cowpea (Difo et al. 2015). The reduction in fat, particularly in soybeans, suggests altered lipid metabolism and potential microbial lipolytic activity. In contrast, several studies have reported an increase in crude fat content following SF (Igbabul et al. 2014; Olagunju et al. 2018; Oluseyi and Temitayo 2015). This observed increase in fat content may be attributed to the production of fungal fatty acids during SF. Fungi produce fatty acids at varying levels during solid‐state fermentation (Higashiyama et al. 2002).

#### 4.2.5 Total Carbohydrate

Given that carbohydrates are the primary raw materials for most fermentations, their levels decrease post‐fermentation as fermenting microbes utilize them. Various studies have reported a decline in carbohydrate content after fermentation, including in sorghum flour (Ogodo et al. 2019), maize flour (Ogodo et al. 2017), and millet flour (Akinola et al. 2017). The observed significant increase in total carbohydrates in the soybean sample is consistent with prior studies on some legumes, which found that fermentation increased carbohydrate levels (Farinde et al. 2018; Ijarotimi and Ruth Esho 2009). Atuna et al. (2025b) reported the dominance of *Wesseilla confusa*, *Limisilactobacillus* spp., *Enterococcus faecium* , and *Pediococcus acidilactis* in the spontaneous fermentation of maize, sorghum, millet, and soybean. In cereals, the total carbohydrate content decreases because starch hydrolysis is followed by rapid microbial consumption for acid production, with exopolysaccharide synthesis insufficient to offset the loss. However, in soybean flour, the carbohydrate content increased because oligosaccharide hydrolysis and microbial exopolysaccharide biosynthesis generated new soluble fractions that exceeded the consumption. This contrast highlights the role of substrate composition in determining whether microbial metabolism leads to carbohydrate depletion or enrichment.

#### 4.2.6 Total Energy

The observed minimal impact of solid‐state fermentation (SF) on the calorific values of most crops suggests that the fermentation process preserves the overall energy content of these substrates. The notable exception is soybeans, which exhibited a significant 6.2% reduction in calorific value post‐fermentation. This decrease could be attributed to the metabolic activity of fermentative microorganisms that utilize certain macronutrients, particularly lipids or carbohydrates, as energy sources, thereby reducing the net energy available in the fermented product. Conversely, the plantain matrix demonstrated a slight increase (1.3%) in calorific value after fermentation, indicating a possible enhancement in energy density. This increase might result from biochemical transformations during fermentation, such as the breakdown of complex carbohydrates into simpler, more energy‐rich compounds, or concentration effects due to moisture loss. These contrasting responses highlight the substrate‐specific nature of SF and underscore the importance of considering the biochemical composition and fermentability of different crops when evaluating fermentation outcomes. Overall, the data imply that SF can be employed without substantial loss of calorific value for most crops, with soybeans requiring particular attention due to their sensitivity to reductions in energy content during fermentation.

#### 4.2.7 Simple Sugars

The sugar transformation patterns, particularly the divergent glucose responses in plantain and millet, underscore the complex interplay between the indigenous microbiota and substrate composition. Atuna et al. (2025b) reported the dominance of *Wesseilla confusa*, *Pediococcus acidilactis*, and *Enterococcus faecium* in millet, and *Fructobacillus fructosus* , *Fructobacillus tropaeoli* , *Apilactobacillus* sp., together with *W. confusa* in plantain. In millet, this microbial consortium may have promoted starch hydrolysis, leading to a marked increase in maltose (~76%) and elevated glucose levels after fermentation. However, in plantains, the fructophilic metabolism of *Fructobacillus* species drove rapid glucose utilization, explaining the decline in glucose levels after fermentation despite its initially high levels. Across all substrates, sucrose levels remained low due to limited availability and rapid microbial consumption.

However, the exponential increase in sucrose following SF in sweetpotato substrates may be due to the enzymatic hydrolysis of starch and polysaccharides into soluble sugars (Kim et al. 2025).

#### 4.2.8 Minerals

The increase in the Ca content in soybean, Fe content in sorghum, and Zn content in millet, soybean, and sweetpotato following SF in the current study is consistent with previous studies reporting improved mineral (Ca, Fe, and Zn) content after the fermentation of cereal‐based products (Asres et al. 2018; Srivastava et al. 2020). The increased mineral content following SF has been attributed to the loss of dry matter as microorganisms break down carbohydrates and proteins (Day and Morawicki 2018). In a recent systematic review by Atuna et al. (2025a), a positive size effect was observed for iron and zinc in most cereal‐based matrices, indicating that SF positively impacted the mineral concentrations in cereal samples. On the contrary, the decline in calcium and zinc observed in cassava flour agrees with the findings of Aloys and Zhou (2006) and Lazarte et al. (2015), who reported reduced levels of calcium, zinc, and iron in cassava. The reduction in mineral content has been ascribed to the absorption and incorporation of minerals into the microbial biomass (Joshi et al. 2018). These bound minerals may not be released into the edible portion, leading to a perceived decrease in mineral content of the edible portion.

#### 4.2.9 Antinutrient

The remarkable 6.2‐fold increase in TPC observed specifically in sweetpotato flour following SF in the current study aligns with earlier reports of TPC enhancement in other fermented matrices, such as millet koji (Salar et al. 2016) and whole‐grain maize (Salar et al. 2012). The observed increase in TPC is often attributed to the mobilization of phenolic compounds from their bound form to a free state through enzymes produced during fermentation, as reported by Salar et al. (2016). Most plant‐based phenolics are conjugated to sugars as glycosides or other moieties (Vattem and Shetty 2002). *β‐Glucosidase* can hydrolyse phenolic glucosides to release extractable free phenolics, such as aglycones, thereby increasing the phenolic content (Lee et al. 2007). Notably, reduced TPC has been reported following fermentation of cassava, sorghum, and soybean, although no statistical differences were observed in this study. The decline in TPC post‐fermentation has been attributed to microbial enzymatic degradation, oxidative conversion, and binding interactions that lower extractable phenolic levels (Kumar et al. 2025).

The reduction in phytic acid content following SF is consistent with several studies (Atuna et al. 2021; Ejigui et al. 2005; Eltayeb et al. 2007; Kayodé et al. 2007; Osman 2004) that have demonstrated that fermentation is a potent strategy for reducing phytic acid content in plant‐based matrices. The considerable reduction in phytic acid in plant‐based substrates after fermentation has been attributed to the favorable conditions created for endogenous phytase activity (Ntso et al. 2016). Specifically, the optimal pH for endogenous phytase has been reported to range from 4.0 to 5.5 (Afify et al. 2011; De Angelis et al. 2003). Atuna et al. (2025b) observed similar pH ranges (2.9 to 5.1) during the SF of these plant‐based substrates. Phytates are naturally occurring compounds found mainly in whole grains, legumes, nuts, and seeds (Vats and Banerjee 2004). These compounds chelate essential minerals such as iron, zinc, and calcium, thereby reducing their bioavailability in the human body (Egli et al. 2004). Their presence could be linked to high levels of micronutrient deficiencies and malnutrition among preschoolers in sub‐Saharan Africa.

## 5 Conclusion

This study has demonstrated that spontaneous fermentation (SF) plays a crucial role in modifying the techno‐functional and biochemical properties of flours from Ghanaian maize, millet, sorghum, soybean, sweetpotato, cassava, and plantain. These modifications enhanced the suitability of these flours for complementary food formulations by improving nutrient utilization and functional performance. The observed crop‐specific variability in SF's effects highlights the necessity for tailored combination and formulation approaches to maximize nutritional benefits and functional quality. SF's capacity to reduce antinutrients such as phytic acid is particularly significant, as it supports improved mineral uptake, which is essential for infant and young child nutrition. Overall, SF represents a promising, targeted intervention to develop nutrient‐dense complementary food ingredients adapted to the unique properties of indigenous crops.

## Author Contributions

**Richard Atinpoore Atuna:** conceptualization, investigation, methodology, validation, visualization, software, formal analysis, data curation, project administration, writing – original draft, writing – review and editing. **Fortune Akabanda:** conceptualization, methodology, supervision, project administration, writing – review and editing, funding acquisition, validation, investigation, resources. **Jan Makurat:** methodology, funding acquisition, visualization, writing – review and editing, data curation, supervision, resources, software, project administration. **Ursula Bordewick‐Dell:** methodology, validation, funding acquisition, writing – review and editing, formal analysis, data curation, supervision, resources. **Guido Ritter:** methodology, validation, writing – review and editing, software, formal analysis, project administration, data curation, supervision, resources, funding acquisition.

## Funding

During the course of the study, RAA received a scholarship from the German Academic Exchange Service (DAAD) under the programme “Bi‐nationally Supervised Doctoral Degrees” (Programme‐ID: 57693451). Additional support for this research was provided by internal funding from the Münster University of Applied Sciences through the ForschungsStarter programme.

## Ethics Statement

The authors have nothing to report.

## Conflicts of Interest

The authors declare no conflicts of interest.

## Data Availability

The data that support the findings of this study are available on request from the corresponding author. The data are not publicly available due to privacy or ethical restrictions.
